# Enhancing Colorectal Cancer Radiation Therapy Efficacy using Silver Nanoprisms Decorated with Graphene as Radiosensitizers

**DOI:** 10.1038/s41598-019-53706-0

**Published:** 2019-11-19

**Authors:** Khaled Habiba, Kathryn Aziz, Keith Sanders, Carlene Michelle Santiago, Lakshmi Shree Kulumani Mahadevan, Vladimir Makarov, Brad R. Weiner, Gerardo Morell, Sunil Krishnan

**Affiliations:** 1 Department of Radiation Oncology, The University of Texas MD Anderson Cancer Center, Houston, TX 77030 USA; 2 Department of Biology, University of Puerto Rico -Rio Piedras Campus, San Juan, PR 00925-2537 USA; 3 Molecular Sciences Research Center, University of Puerto Rico, San Juan, Puerto Rico 00926-2614 USA; 4 Department of Physics, University of Puerto Rico -Rio Piedras Campus, San Juan, PR 00925-2537 USA; 5 Department of Chemistry, University of Puerto Rico -Rio Piedras Campus, San Juan, PR 00925-2537 USA; 6 Comprehensive Cancer Center, University of Puerto Rico, San Juan, PR 00936-3027 USA; 7 Present Address: Department of Radiation Oncology, Mayo Clinic Florida, Jacksonville, FL 32224 USA

**Keywords:** Nanostructures, Nanotechnology in cancer

## Abstract

Metal nanoparticles have significant interaction cross-sections with electromagnetic waves due to their large surface area-to-volume ratio, which can be exploited in cancer radiotherapy to locally enhance the radiation dose deposition in tumors. We developed a new type of silver nanoparticle composite, PEGylated graphene quantum dot (GQD)-decorated Silver Nanoprisms (pGAgNPs), that show excellent *in vitro* intracellular uptake and radiosensitization in radiation-sensitive HCT116 and relatively radiation-resistant HT29 colorectal cancer cells. Furthermore, following biodistribution analysis of intravenously injected nanoparticles in nude mice bearing HCT116 tumors radiosensitization was evaluated. Treatment with nanoparticles and a single radiation dose of 10 Gy significantly reduces the growth of colorectal tumors and increases the survival time as compared to treatment with radiation only. Our findings suggest that these novel nanoparticles offer a promising paradigm for enhancing colorectal cancer radiation therapy efficacy.

## Introduction

Cancer is the second leading cause of mortality in the United States and, despite advances in prevention and early detection, colorectal cancer (CRC) is the third leading cause of cancer-related mortality in males and females in the United States^1^. Traditionally, CRC is treated with surgery in the early stages, while a combination of preoperative chemoradiation therapy (CRT) and surgery is used in the more common locally advanced stages. Preoperative CRT results in only about 15% of patients achieving a complete pathological response, *i.e*., no viable tumor remains within the surgical specimen at the time of surgery^2,3^. In an attempt to increase this meager percentage, higher radiation doses can be used to improve tumor downstaging and local control of tumors^3^. However, the dose escalation needed to further improve the complete pathological response rates also increases the risk of toxicity and exceeds the tolerance of adjacent healthy tissues^3^. A better alternative may be to combine standard-dose radiotherapy with radiosensitizers to enhance the radiation therapy efficacy locally within tumors while sparing adjacent healthy tissues^4–9^.

Nanoparticles have generated excitement in cancer research due to their unique properties that promise to enhance the efficacy of conventional cancer therapy^6–26^. Several studies indicate that nanoparticles have a significant interaction cross-section with biomolecules and electromagnetic waves due to their large surface area-to-volume ratio, which coupled with their enhanced accumulation in tumors due to leaky vasculature and the possibility of even greater uptake via tumor/organ-specific targeting, makes nanoparticles excellent candidates for radiosensitization^9,13,15^. In radiotherapy, the enhanced interaction cross-section of high atomic number (Z) metallic nanoparticles with ionizing radiation leads to greater radiation dose deposition in tumors, which results in potent radiosensitization^7–9,15^.

Metal based nanoparticles, such as gold and silver, have been evaluated pre-clinically in *in vitro* and *in vivo* studies^6–14,20^. Previous reports suggest that maximum radiosensitization occurs at lower beam energies (≤1 MeV), when the photoelectric effect is dominant and is strongly dependent on the Z of the radiosensitizing material^7,9,14^. This interaction of photons with tumor-localized high-Z particles results in greater ionization, greater generation of secondary electrons and free radicals, and ultimately, greater DNA damage^7^. Some studies note that radiosensitization is possible even with clinical beams of energies lower than 6 MeV, where the dominant interaction is Compton scattering, which is less dependent on Z^6,7,9^. This is likely due to the poly-energetic behavior of these clinical beams that may include photons at lower energies, especially at the depths where the tumor resides^7,9^. Among metal based nanomaterials, gold nanoparticles (GNPs) have received the greatest attention and are considered a benchmark in radiotherapy due to their attractive properties, such as good biocompatibility, chemical stability, ease of surface modification, and high X-ray absorption coefficients. In a study by Shi *et al*., the authors reported an enhancement in the radiosensitivity of HCT116 human CRC cells treated with tiopronin-coated GNPs (Tio-GNPs) combined with a low-energy X-ray (26 keV effective energy) source^20^. The authors compared intravenous and intratumoral injection of Tio-GNPs into HCT116 tumors in mice and found that treatment is only effective when particles are injected intratumorally. Other metal-based nanoparticles such as hafnium oxide^19^, silver^10,12,22^, and iron oxide^23^, have also been studied to enhance the therapeutic efficiency of radiotherapy. A recent study by Liu *et al*. reported that spherical silver nanoparticles (AgNPs) have a higher radiosensitizing activity than GNPs in the treatment of malignant glioma due to their radiosensitizing and antiproliferative activities^22^. The authors found that the combination of AgNPs and radiotherapy has significantly enhanced the mean survival time in glioma-bearing rats as compared to similar molar or mass concentration of GNPs combined with radiotherapy. Despite the large number of publications pointing at the effectiveness of metal nanoparticles as radiosensitizers, there are still many unknowns when it comes to defining the ideal formulation of nanoparticles to enhance the therapeutic efficacy of radiotherapy. Proposed strategies rely on the optimization of the shape, size, physicochemical properties, surface targeting ligands of these metal-based nanoparticles^11,20,24^, or the use of hybrids or composites^25^. For example, Ma *et al*.^26^ suggested that the shape of metal-based nanoparticles, such as gold, has a significant influence on cancer radiotherapy due to their cellular uptake and radiosensitization effects, while Habiba *et al*.^16^ showed that the decoration of silver nanoparticles with graphene enhance the cellular uptake and biocompatibility of silver. Here, we report radiosensitization effects of silver nanoprisms decorated with PEGylated graphene quantum dots (pGAgNPs) in CRC *in vitro* and *in vivo*. We hypothesize that decorating silver nanoprisms with PEGylated graphene quantum dots (pGQD) helps maintain their anisotropic prismatic shape and increases their cellular internalization within tumors, both of which contribute to enhanced radiosensitization in CRC.

## Materials and Methods

### Synthesis of nanoparticles

#### Synthesis of bare silver nanoprisms (AgNPs) and PEGylated silver nanoprisms (pAgNPs)

For AgNPs synthesis, we followed the method reported previously by Zhang *et al*. with a minor modification^27^. Briefly, we prepared a reaction mixture by adding (500 μL, 10 mM) of silver nitrate (Sigma-Aldrich Co., St Louis, MO, USA), (1.5 mL, 30 mM) of trisodium citrate (Sigma-Aldrich Co., St Louis, MO, USA), and H_2_O_2_ (60 μL, 30 wt.%) into 25 mL nanopure water and stirred vigorously for 5 min at room temperature. Then, (500 mL, 50 mM) of sodium borohydride (Sigma-Aldrich Co., St Louis, MO, USA) was rapidly injected into this reaction mixture, creating a light-yellow solution that changed color to a deep-yellow, followed by red, green, and eventually blue within 5 min. The resulting AgNP solution was stored at 4 °C for future use.

For pAgNPs synthesis, thiol-PEG-NH_2_ (3 mL, 5 kDa, 300 μM) and (30 μL, 20 mM) K_2_CO_3_ (Sigma-Aldrich Co., St Louis, MO, USA) were added to 60 mL AgNPs solution to yield a new solution with a concentration of 16 μg/mL. The solution was stirred gently for 2 h at room temperature and excess PEG was removed using 100 kDa centrifugation dialysis tubes. The resulting pAgNP solution was washed with nanopure water and centrifuged three times at 14,000 rpm for 20 min. Finally, the supernatant containing remnant silver ions or small particles was discarded to yield the final pAgNP solution.

#### Synthesis of PEGylated GQDs (pGQDs)

For pGQDs synthesis, we used a bottom-up synthesis approach as described previously by Habiba *et al*.^16,28^. Briefly, a mixture of nickel oxide powder (0.25 wt.%, Alfa Aesar), bis (3-aminopropyl) terminated PEG (1.25 wt.%, Sigma–Aldrich), and benzene (98.5 wt.%, Sigma–Aldrich) was prepared, and then irradiated for 45 min with a 1,064 nm pulsed Nd:YAG laser (Continuum Surelite II, KDP doubling crystal, 10 Hz, 10 ns pulse width). The pGQD solution was centrifuged to separate it from benzene and precipitated nickel oxide, followed by vacuum evaporation. Finally, the dry pGQDs were re-suspended in nanopure water and purified using dialysis bags with a molecular weight cut-off of 6–8 kDa (Spectrum Labs, USA).

#### Synthesis of pGAgNPs

GQD-decorated PEGylated Silver Nanoprisms (pGAgNPs) were fabricated via non-covalent electrostatic interaction between the negatively charged citrate-capped AgNPs and the positively charged pGQDs similar to the approach described previously with a minor modification^28,29^. We added AgNPs (3.5 mL, 130 μg/mL) to pGQDs (1.5 mL, 1 mg/mL) in a total volume of 15 mL nanopure water and stirred the solution for 2 h at room temperature. The solution was then purified from excess free pGQDs using 100 kDa centrifuge dialysis tubes and washed with nanopure water two times. Finally, the pGAgNPs solution was centrifuged at 14,000 rpm for 20 min and the supernatant was discarded to obtain a narrow size distribution of pGAgNPs.

#### Nanoparticle characterization

Transmission electron microscopy (TEM) images were obtained using an electron microscope (JEOL JEM1010, Japan), operated at 80 kV. UV-Vis spectra were carried out using a double beam spectrophotometer (Varian Cary 100 Bio, Agilent, CA). The hydrodynamic size and zeta potential were obtained using a Zetasizer (Malvern Zetasizer Nanoseries, UK). The nanoparticle size distribution was analyzed from 15 TEM images for over 500 nanoparticles using ImageJ.

### In vitro

#### Cell culture

Human CRC cell lines HCT116 and HT29 were obtained from the American Type Culture Collection. All cells were grown in McCoy’s 5 A modified medium (GE Healthcare Life Sciences) supplemented with 10% fetal bovine serum (Sigma Aldrich), (100 unit/mL) penicillin, and (100 mg/mL) streptomycin (Life Technologies) and grown in a 37 °C incubator containing 5% CO_2_.

#### Cellular uptake of nanoparticles

The HCT116 cells were plated in 8-well glass chamber slides (Nunc Lab-Tek) at a density of 30,000 cells per well and grown overnight. The following day, cells were treated with fresh media, pAgNPs, or pGAgNPs diluted in media (7 µg/mL) and incubated for 24 h. Afterwards, cells were washed three times with PBS to remove non-internalized nanoparticles and then fixed with ice cold methanol. Nuclei were then stained with Hoechst 33258 (2 µg/mL) for 10 min at room temperature in a dark room, washed with PBS, and imaged using the Cytoviva Hyperspectral Imaging system in dark field mode to image the high scattering of light by the nanoparticles. Each group was prepared in duplicate and images from different fields were obtained. The reflectance spectrum of the internalized nanoparticles was acquired at 5 spots in 5 different fields to confirm the presence of internalized nanoparticles. The experiments were performed independently two times.

The cellular uptake of nanoparticles was validated and quantified using inductively coupled plasma mass spectrometry (ICP-MS, Agilent 7900 ICP-MS). HCT116 cells were plated at a density of 30,000 cells per dish in 30 mm petri dishes, treated with complete media, pAgNPs, or pGAgNPs (7 µg/mL), and incubated for 24 h. Afterwards, cells were washed three times with PBS, trypsinized, counted, centrifuged, and digested in concentrated nitric acid and the elemental silver concentration was estimated by ICP-MS. The ICP-MS experiments were performed three times independently, each in triplicate.

#### Cell proliferation assay

We plated 6,000 HCT116 and HT29 cells each in separate 96-well plates and grew them overnight. The following day, cells were treated with fresh media containing nanoparticles (AgNPs, pAgNPs, or pGAgNPs) at concentrations of 0, 2.5, 5, 10, 17 or 20 µg/mL and incubated for 24 h. Afterwards, cells were washed with fresh media and 20 µl MTS CellTiter 96® Aqueous Solution Cell Proliferation Assay (Promega, USA) were added to each well and incubated at 37 °C for a minimum of 1 h. Before measuring the absorbance of treated cells, background absorbance was first subtracted using a set of wells containing media only, normalized, and expressed as a relative percentage of the plate-averaged cells treated with nanopure water control. Absorbance was then measured at 490 nm on a microplate reader (Cytation 5, Biotek, VT). All measurements were made according to manufacturer’s instructions.

#### Radiosensitization

HCT116 or HT29 cells were plated at a density of 30,000 cells per dish in 60 mm cell culture dishes and incubated until they reached 60–70% confluence. Cells were treated with AgNPs or pGAgNPs (17 µg/mL) and incubated for 24 h. A group of cells treated with media served as control. Afterwards, cells were washed twice with PBS and irradiated using the XRAD 320 orthovoltage irradiator (Precision X-Ray, Inc.), operating at 320 kVp and 12.5 mA at doses of 0, 2, 4, or 6 Gy. Immediately after irradiation, cells were trypsinized, counted, diluted, and reseeded in 6-well plates. Cells were incubated for ten days and then fixed and stained with 0.5% crystal violet diluted in ethanol. All colonies containing 50 or more cells were counted, and the plating efficiency (PE) for each cell line was determined. The surviving fraction for each group was calculated as the average number of colonies divided by the product of the number of cells plated and PE, and plotted on a log scale against irradiation doses. The experiments were performed independently at least three times, each in 6 replicate.

#### Immunofluorescence staining of 53BP1

Cells were plated onto four 8-well chamber coverslips at a density of 1 × 10^5^ cells per well and then incubated overnight. Cells were treated the next day with complete media, 17 µg/mL of pAgNPs or 17 µg/mL of pGAgNPs and incubated for 24 h. Afterwards, cells were washed two times with PBS and irradiated with a dose of 2 Gy using the XRAD 320 orthovoltage irradiator (320 kVp and 12.5 mA). A group of cells that were not irradiated served as control. We fixed the cells 0.5, 3, or 24 h after irradiation with ice cold methanol for 10 min, permeabilized with 0.05% Tween-20, and washed with PBS. The immunofluorescence staining of the cells was performed by blocking them first with 1% BSA for 1 h at room temperature, and then incubating them with 53BP1 antibody (Cell Signaling, Colorado, USA) at a dilution of 1:200 in 1% BSA for 1 h at room temperature. Next, all slides were washed and rinsed three times with PBS before being incubated with a mixture of Hoechst 33258 (2 µg/mL) and Alexa Fluor 488 goat anti-rabbit secondary antibody (Cell Signaling, Oregon, USA) at a dilution of 1:300 in 1% BSA for 1 h at room temperature. Finally, the coverslips were washed three times with PBS, chamber walls collapsed, and imaged on a microscope Cytation 5 at 40x magnification. Individual foci per nucleus were counted manually and plotted over time for each of the groups. The experiments were performed independently two times, each in triplicate. All data were analyzed from at least 10 different images for each group.

#### Reactive oxygen species (ROS) measurement

Cells were plated at a density of 1 × 10^5^ in black-wall 96-well plates, incubated for 24 h, and then treated and incubated for 24 h with pAgNPs or pGAgNPs at a concentration of 17 µg/mL. Cells treated with complete media served as a control. After incubation, cells were washed with PBS two times and the ROS detection reagent was added using a Total Reactive Oxygen Species Assay Kit (Invitrogen, Fisher Scientific Pittsburgh, PA, USA). Cells were then incubated in a 37 °C incubator with 5% CO_2_ for 60 min. Next, cells were irradiated with a dose of 6 Gy and protected from light before measuring the fluorescence according to the manufacturer’s instructions. A group of cells that were not irradiated served as no radiation control. The experiments were performed independently two times, each in triplicate.

### In vivo

#### Animal tumor model

Male Swiss nu/nu mice aged 5–8 weeks upon acquisition from The University of Texas MD Anderson Cancer Center’s Experimental Radiation Oncology Mouse Facility were handled following approved Institutional Animal Care and Use Committee (IACUC) protocols. 1 × 10^6^ HCT116 cells suspended in 50 µL PBS were injected subcutaneously into the right thigh of anesthetized mice. Tumors were left to grow undisturbed until they reached an average of 8 mm in diameter prior to being treated in all *in vivo* experiments.

#### Biodistribution

Nanoparticle biodistribution was assessed in 6 tumor-bearing mice (3 mice in each treatment) 24 h after being treated with pAgNPs or pGAgNPs (70 μg Ag per mouse diluted in 200 μL sterile nanopure water). The accumulation of nanoparticles per organ was determined using ICP-MS by quantifying silver content in specimens obtained from major organs (brain, lung, heart, liver, spleen, gastrointestinal tract, kidney, muscle), blood, and tumors. A portion of each organ was placed in a glass vial, lyophilized, and fully digested twice by first adding 1.5 mL concentrated nitric acid (70%, Optima grade, Fisher Scientific Pittsburgh, PA, USA) and heating at 155 °C for 2 h, and then by adding 1 mL H_2_O_2_ (35%, Optima grade Fisher Scientific Pittsburgh, PA, USA) and heating at 110 °C until almost dry. The digested sample volumes were diluted to a final volume of 10 mL by adding freshly prepared 2% nitric acid before ICP-MS analysis.

#### Radiosensitization

Tumor-bearing mice were randomly divided into 6 groups: control (vehicle treatment without nanoparticles and radiation), radiation alone (RT), pAgNPs treatment alone (pAgNPs), pAgNPs treatment with radiation (pAgNPs + RT), pGAgNPs treatment alone (pGAgNPs), and pGAgNPs treatment with radiation (pGAgNPs + RT). Each group without radiation consisted of 5 mice and each group with radiation consisted of 10 mice for a total of 45 mice. Mice in the nanoparticle treatment groups received a single tail-vein injection of pAgNPs or pGAgNPs (56 μg Ag per mouse diluted in 200 µL sterile nanopure water) and a single dose of 10 Gy radiation using a 250 kVp irradiator (Phillips 250 orthovoltage irradiator) 24 h after nanoparticle treatment. The tumor growth was measured and recorded every three days using a digital caliper and tumor volume was calculated according to the formula: v = πab2/6 where v = tumor volume (mm^3^), a = long axis (mm) and b = short axis (mm). The mean tumor volume for each group was plotted over time until tumors reached a diameter of 1.5 cm in the long axis, at which point mice were euthanized. Survival data were recorded and plotted using Kaplan–Meier techniques with survival time being calculated from the date of nanoparticle treatment to the date of euthanasia for each mouse.

### Statistical analyses

Each *in vitro* experiment was conducted in triplicate and data were summarized as mean ± SD. Differences among groups were analyzed by using one-tailed Student’s t-tests and two-way analysis of variance, as indicated in the captions. For the *in vivo* biodistribution and tumor growth delay data, the mean values and associated standard errors of the mean (SEM) values for each group were calculated and the differences among groups were analyzed using the Mann-Whitney test. For the *in vivo* survival data, the median survival for each group were calculated and differences among groups were analyzed using the long rank test. Statistical significant was defined as *P* < 0.05. We performed all statistical analyses with GraphPad Prism software (GraphPad Software Inc., La Jolla, CA).

### Ethical conduct of experiments

Permission from the Institutional Review Board at MD Anderson Cancer Center was received before commencing any animal experiments. All experiments have been conducted following the Institutional Animal Care and Use Committee (IACUC) protocols.

## Results and Discussion

### Nanoparticle preparation and characterization

**Figure 1:**
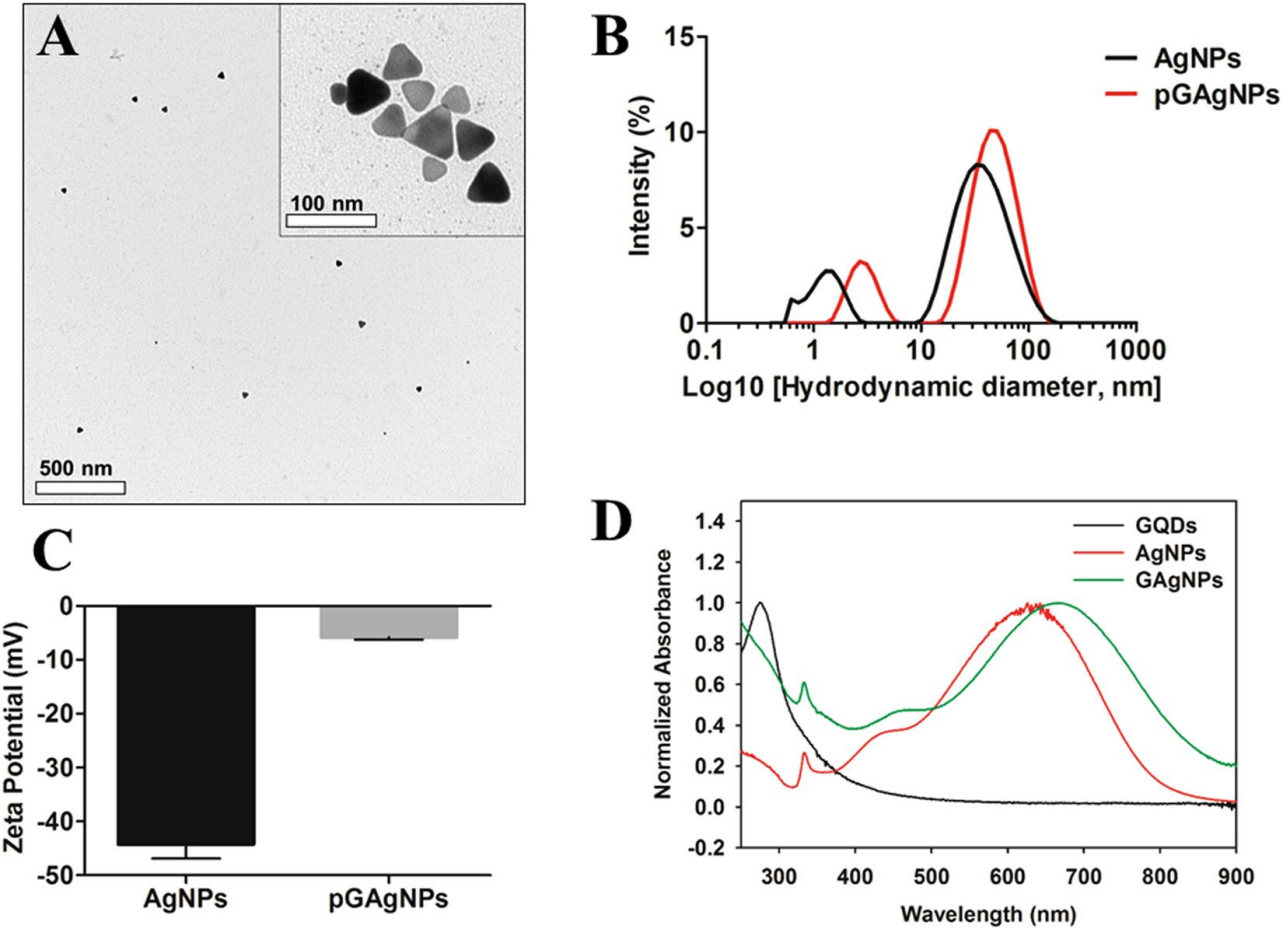
(**A**) Characterization of pGAgNPs using transmission electron microscopy; (**B**) Characterization of AgNPs and pGAgNPs using dynamic light scattering. The spectra show a redshift in the particle size distribution upon the decoration of AgNPs with pGQDs; (**C**) Zeta potential of AgNPs and pGAgNPs, showing a decrease in charge after decoration with pGQDs; (**D**) Characterization of pGQDs, AgNPs, and pGAgNPs using UV-Vis spectroscopy.

Bare silver nanoprisms (AgNPs) were synthesized following the method published by Zhang *et al*. with a minor modification^27^. Afterward, AgNPs were coated with PEGylated graphene quantum dots (pGQDs), synthesized using a similar approach that we reported previously^16^. To elucidate whether pGQDs offer a beneficial functionality to AgNPs from the material science and radiotherapy standpoints, we prepared PEGylated silver nanoprisms (pAgNPs) for comparison. For PEGylation of AgNPs, an amino terminated polyethylene glycol (PEG) with a similar molecular weight as GQDs was selected to prepare pAgNPs. The pAgNPs and pGAgNPs were characterized by TEM, UV-Vis spectroscopy, and zetasizer. A representative TEM image of pGAgNPs in Fig. 1A shows anisotropic triangular-shaped nanoparticles, and the inset image at high magnification shows particles with a size range of 18–45 nm. Meanwhile, we found that PEGylation of AgNPs to produce pAgNPs changes the triangular shape of the prisms into quasi-triangular or circular discs of silver (Fig. S1). This transformation of the triangular shape of pAgNPs has been reported previously by Liu *et al*.^30^. They indicated that due to the high affinity of silver to thiols, the Ag surface is etched through a catalytic redox process, in which the atoms in the oxidized nanoprism tips are removed and form silver thiolate complexes. Therefore, the presence of pGQDs used to decorate AgNPs is most likely protecting their anisotropic shape and preserving their plasmonic properties.

The hydrodynamic size of bare AgNPs and pGAgNPs obtained by dynamic light scattering (DLS) spectra and measured by the zetasizer is shown in Fig. 1B. The size distribution of both particles is composed of two narrow distribution peaks, one around 1.4 and 3 nm and another centered around 43 and 52 nm for AgNPs and pGAgNPs, respectively. The major peaks in both pGAgNPs spectra represent their real hydrodynamic size distribution, which is close to their size distribution obtained by TEM in Fig. 1A. The minor peaks could be due to rotational diffusion, which has been observed in similar two-peak size distributions reported in studies of other anisotropic nanoparticles (*e.g*., gold nanorods and nanoprisms)^31,32^. For comparison with pAgNPs, we observed that the peaks corresponding to their hydrodynamic size were centered around 7.5 and 68 nm (see Fig. S2A). Figure 1C shows that the zeta potential of pGAgNPs is −5.8 mV as compared to −44 mV of bare AgNPs after decoration of AgNPs with pGQDs. The difference in the zeta potential and hydrodynamic sizes between AgNPs and pGAgNPs suggests pGQD capping of AgNPs masks and neutralizes their surface charge. The zeta potential of pAgNPs changed from −44 mV to 16.6 mV after PEGylation (see Fig. S2B). The larger hydrodynamic size of pAgNPs compared to their size in TEM is likely due to the solvation layers on their surface or low aggregation^33^.

Figure 1D shows the UV-Vis spectrum of GQDs with a maximum absorption at 275 nm, which is consistent with the absorption spectrum of GQDs^34^, and likely corresponds to π–π* transition of aromatic sp2 C domains^16,28^. In the UV-Vis absorption spectra of AgNPs and pGAgNPs shown in Fig. 1D, the weak and narrow peak at 330 nm is attributable to the out-of-plane dipole and corresponds to oscillations of the metal surface electrons along the nanoparticle thickness. In the absorption spectra of AgNPs, the second broad band at 400–550 nm is attributable to the in-plane quadrupole and the intense band at 630 nm is attributable to the in-plane dipole^35,36^. In the absorption spectra of pGAgNPs, the in-plane dipole absorption band is red-shifted to 667 nm after decoration with pGQDs and a prominent absorption band corresponding to pGQDs is noted at approximately 275 nm. This change in the spectrum is indicative of successful decoration of the AgNPs with pGQDs.

### In vitro

#### Cellular uptake of nanoparticles

**Figure 2:**
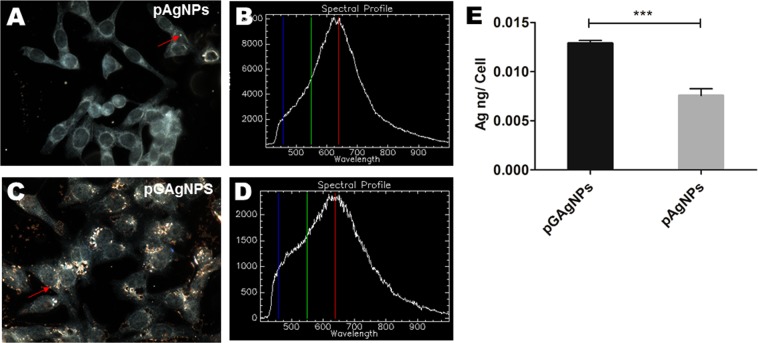
(**A**–**D**) Hyperspectral imaging in dark field mode and the corresponding spectral mapping of HCT116 cells treated with pAgNPs (**A**,**B**) and pGAgNPs (**C**,**D**). The images show higher cellular uptake of pGAgNPs compared to pAgNPs; (**E**) ICP-MS analysis of HCT116 cells treated with pAgNPs or pGAgNPs reveals nearly 1.7-fold increase in cellular uptake of pGAgNPs compared to pAgNPs. Comparisons between groups was by Student’s t-test statistical analysis *P*-values obtained are as follows: *P < 0.05 **P < 0.01 ***P < 0.001.

To investigate the cellular uptake of the pAgNPs and pGAgNPs qualitatively in HCT116 cells, we utilized the CytoViva Hyperspectral Imaging (HSI) system in dark-field mode. In Fig. 2A, the dark field image of cells treated with pAgNPs shows the presence of a few bright spots. Figure 2B displays the corresponding HSI signal from the selected region (red arrow) in Fig. 2A and shows internalized pAgNPs. Figure 2C clearly shows more bright spots resulting from the cellular internalization of pGAgNPs in the cells. Figure 2D displays the corresponding spectrum of the selected area (red arrow) in Fig. 2C, which is consistent with the UV-Vis absorption spectrum of pGAgNPs shown in Fig. 1D, again confirming the presence of pGAgNPs within cells. The intensity (y-axis) does not necessarily indicate that the overall uptake in cells is high or low as the spectra were taken at some points and the signal intensity varies greatly with the z-axis focus. While the qualitative spectra help to verify that the bright spots correspond to the nanoparticles in HCT116 cells, the extent of cellular uptake was quantified by ICP-MS measurements of elemental silver concentration within the cells (Fig. 2E). The cellular uptake of pGAgNPs (0.0135 ng of Ag/cell) was significantly greater than that of pAgNPs (0.0076 ng of Ag/cell), as shown in Fig. 2E. Zhou *et al*. showed previously that A549 human lung carcinoma cells internalized less bare silver or gold nanoparticles than graphene oxide/silver or graphene oxide/gold nanocomposites^17^, and suggested that graphene enhances the cellular uptake when combined with metals in nanocomposites. These results indicate that pGQDs play a critical role in the internalization of silver nanoparticles.

#### Cell proliferation

Before assessing the efficacy of pGAgNPs in sensitizing HCT116 and HT29 cells to radiation, we first evaluated the stand-alone toxicity of the nanoparticles in cell proliferation assays. The nanoparticles (AgNPs, pAgNPs and pGAgNPs; see Fig. S3) do not significantly change the viability of either cell line across a range of concentrations.

#### Radiosensitization

**Figure 3:**
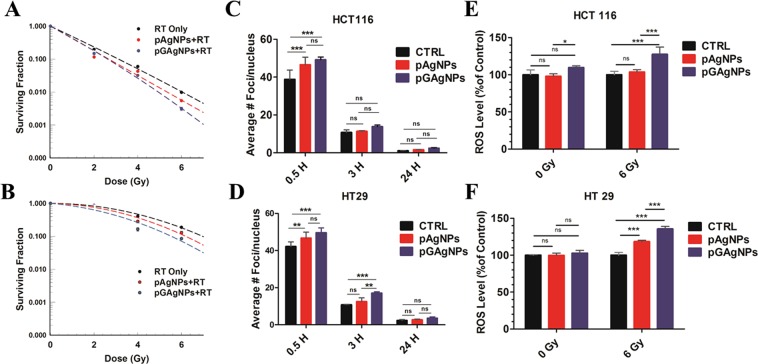
(**A**,**B**) Cell survival fraction of HCT116 (**A**) and HT29 (**B**) cells treated with RT, pAgNPs + RT, or pGAgNPs + RT; (**C**,**D**) Analysis of 53BP1 immunofluorescence foci of irradiated control cells and cells treated with pAgNPs or pGAgNPs 0.5, 3, and 24 h after irradiation; (**E**,**F**) ROS level analysis of irradiated RT only and cells treated with pAgNPs or pGAgNPs. Comparisons between groups was by two-way ANOVA *P < 0.05 **P < 0.01 ***P < 0.001.

We then proceeded to evaluate radiosensitization using classical *in vitro* clonogenic assays in radiation-sensitive HCT116 and relatively radiation-resistant HT29 cells. Tumor cell survival curves displayed in Fig. 3A,B demonstrate a mild increase in the radiosensitization with pGAgNPs than with pAgNPs. The dose enhancement factor at 10% surviving fraction (DEF_10_) was determined by dividing the radiation dose in the absence of the radiosensitizer (*i.e*., drugs or nanoparticles) by the radiation dose in presence of the radiosensitizer to reduce the tumor cell survival fraction to 10% in the absence of radiosensitizers to that at which the surviving fraction is 10%. The DEF_10_ in HCT116 cells were 1.18 and 1.2 for pAgNPs and pGAgNPs, whereas they were 1.125 and 1.28, respectively, in HT29 cells.

#### Immunofluorescence staining of 53BP1

The dominant and proximate cause of radiation-induced cytotoxicity is DNA damage. We analyzed DNA damage in irradiated non-treated cells (*i.e*., control), and cells treated with pAgNPs, or pGAgNPs by immunofluorescence staining of 53BP1, a protein that binds to DNA strand breaks and initiates DNA repair^37^. Unrepaired DNA strand breaks are marked by the persistence of 53BP1, visualized as fluorescent foci in the nucleus (Figs. S4 and S5). Figure 3C,D show the average number of foci persisting 0.5, 3 and 24 h after irradiating HCT116 and HT29 cells with a dose of 2 Gy. As seen in Fig. 3C, treatment of the radiation sensitive HCT116 cells with pGAgNPs or pAgNPs significantly increased radiation-induced DNA damage at the 0.5 h time point. However, no significant difference was observed at the 3 and 24 h time points and the cells were able to repair the damage induced by the 2 Gy dose. Similarly, in HT29 cells (Fig. 3D) pGAgNPs treatment significantly increased DNA damage over control at the 0.5 and 3 h time points and over pAgNPs at 3 hr, whereas pAgNPs treatment did not result in increased DNA damage at the 3 and 24 h time points. This difference between RT only and treatment with either pGAgNPs or pAgNPs-mediated augmentations of radiation-induced DNA damage after 0.5 h may be due to the higher radiosensitization of both nanoparticles and contributing to a greater degree of DNA damage induced within cells.

#### Reactive oxygen species (ROS) measurement

Aside from DNA damage, another ubiquitous consequence of radiation is ionization of atoms and generation of highly reactive free radicals. ROS, the most potent of these free radicals, can travel to and indirectly damage DNA as opposed to radiation directly damaging DNA^38^. We measured total ROS generation in cells and observed a negligible increase in ROS when cells were treated with nanoparticles alone (*i.e*., pAgNPs or pGAgNPs). However, when cells were irradiated with a dose of 6 Gy, there was a significant increase in ROS production in pGAgNP-treated cells, 27% and 35% higher than non-treated groups in HCT116 and HT29 cells, respectively (Fig. 3E,F). The pAgNPs, on the other hand, did not significantly increase ROS production in irradiated HCT116 cells and only minimally increased ROS production, 18% more, in irradiated HT29 cells. This inability of pAgNPs to induce potent ROS production may be due to their low cellular uptake or the detection limits of the assay kit. However, taken together with the results of the clonogenic assay and the 53BP1 immunofluorescence studies, these studies corroborate the ability of pGAgNPs to potentially serve as radiosensitizing agents attributable to their greater cellular internalization.

### In vivo

#### Biodistribution

**Figure 4:**
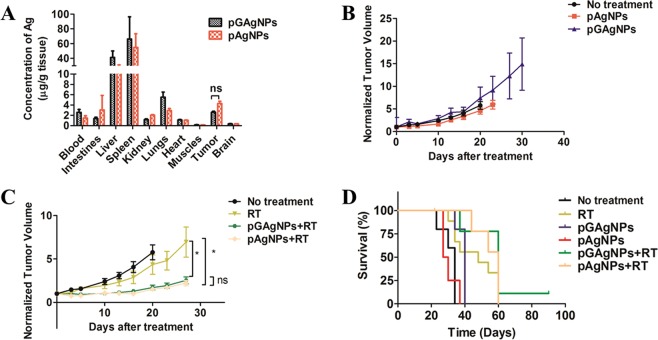
(**A**) Biodistribution analysis by inductively-coupled plasma mass spectrometry of elemental Ag concentration in tumor, blood and normal tissue samples 24 h after intravenous administration *in vivo*; (**B**,**C**) Tumor growth delay curves of treated without RT (**B**) or with RT (**C**,**D**) Kaplan-Meier survival curve of mice. Median survival time for mice was 34, 48, 40, 28.5, 60 and 60 days for no treatment, RT, pGAgNPS, pAgNPS, pGAgNPS + RT, and pAgNPS + RT, respectively. Comparison between groups was by Student’s t-test. *P < 0.05 **P < 0.01 ***P < 0.001.

As a step forward in the process of translating these nanoparticles to the clinic, we validated the *in vitro* radiosensitization results with *in vivo* studies. First, we performed a biodistribution study to measure the distribution and concentration of pAgNPs or pGAgNPs. ICP-MS determination of elemental concentration of silver revealed slightly increased accumulation of pAgNPs than pGAgNPs in tumors, although the difference is not significant (*P* = 0.10) (Fig. 4A). The high accumulation in liver and spleen for both particles is likely related to uptake by hepatic stellate and splenic macrophages, consistent with prior reports^39,40^. The hepatic and splenic uptake of pGAgNPs was higher as compared to pAgNPs, which may be ascribed to the adsorption of proteins on graphene that cause their uptake by macrophage cells.

### Radiosensitization

An *in vivo* tumor growth delay assay was utilized to evaluate radiosensitization effects of the nanoparticles. Treatment with either pGAgNPs or pAgNPs without RT (*P* = 0.14 and 0.98, respectively) does not induce tumor growth delay (Fig. 4B). When pGAgNPs and pAgNPs are combined with RT, tumor growth is inhibited effectively as compared to RT alone (*P* = 0.0468 and 0.0132, respectively; Fig. 4C). However, no significant difference was observed between pAgNPs + RT and pGAgNPs + RT (*P* = 0.4517). On evaluating survival of these mice as shown in Fig. 4D, both pAgNPs and pGAgNPs enhanced median survival (60 days for mice treated with pAgNPs and pGAgNPs compared to 48 days for mice treated with RT alone). However, the difference in median survival was only significant between mice treated with pGAgNPs compared to mice treated with RT alone (*P* = 0.043). Shi *et al*.^20^ injected 732.6 µg and 366.3 µg of GNPs per mouse intravenously and intratumorly, respectively, before using a single dose of 10 Gy. The intravenously injected GNPs and RT group did not show any radiosensitization enhancement as compared to RT alone. Other studies using hafnium oxide nanoparticles as radiosensitizers of HCT116 tumors showed potent tumor inhibition after 25 days and significantly increased survival when tumors containing hafnium oxide nanoparticles were exposed to a single dose of 8 Gy^19^. However, the hafnium oxide nanoparticles in that study were injected intratumorally at a high concentration of 64 g/L. Such an approach may be limited to tumors with well-defined borders, easily accessible by interstitial probes, and located far from critical organs. In contrast, our approach has broader applications and is non-invasive.

## Conclusion

PEGylated GQD-decorated Silver Nanoprisms (pGAgNPs) show better intracellular uptake as compared to PEGylated Silver Nanoprisms (pAgNPs) when both types of nanoparticles were evaluated *in vitro* in radiation-sensitive colorectal cancer cells. This enhanced uptake resulted in slight increase in the radiosensitization effect *in vitro* as well. The increments in ROS and DNA damage associated with these nanoparticles are key contributors to the observed radiosensitization. In nude mice bearing HCT116 tumors, after 50 days of treatment, radiation therapy combined with either pAgNPs or pGAgNPs was found to be ~175% more effective at inhibiting tumor growth in comparison to radiation therapy alone. Although the inhibition of tumor growth was not significant between treatment with pAgNPs + RT and pGAgNPs + RT, the median survival was slightly increased with pGAgNPs + RT. Taken together, our *in vitro* and *in vivo* data suggest that pGAgNPs and pAgNPs radiosensitize tumors and may improve radiation therapy efficacy without the need to increase the radiation doses.

## Supplementary information

Supplementary data

## Data Availability

All data obtained and analyzed in this research are included in this article and its Supplementary Information file.
