# Supplementary material for: Enhancing Colorectal Cancer Radiation Therapy Efficacy using Silver Nanoprisms Decorated with Graphene as Radiosensitizers
Source: Sci Rep. 2019 Nov 19;9:17120. doi: 10.1038/s41598-019-53706-0 (PMC6864075; doi:10.1038/s41598-019-53706-0)
Supplement: Supplementary file 1 — Supplementary data [file 41598_2019_53706_MOESM1_ESM.docx]

**Enhancing Colorectal Cancer Radiation Therapy Efficacy using Silver Nanoprisms Decorated with Graphene as Radiosensitizers**

Khaled Habiba,^1,*^ Kathryn Aziz,^1^ Keith Sanders,^1^ Carlene Michelle Santiago,^1,2,3^ Lakshmi Shree Kulumani Mahadevan,^1^ Vladimir Makarov,^3,4^ Brad R. Weiner,^3,5,6^ Gerardo Morell,^3,4,6^ and Sunil Krishnan^1,*^

1. Department of Radiation Oncology, The University of Texas MD Anderson Cancer Center, Houston, TX 77030, USA

2. Department of Biology, University of Puerto Rico -Rio Piedras Campus, San Juan, PR 00925-2537, USA

3. Molecular Sciences Research Center, University of Puerto Rico, San Juan, Puerto Rico 00926-2614, USA

4. Department of Physics, University of Puerto Rico -Rio Piedras Campus, San Juan, PR 00925-2537, USA

5. Department of Chemistry, University of Puerto Rico -Rio Piedras Campus, San Juan, PR 00925-2537, USA

6. Comprehensive Cancer Center, University of Puerto Rico, San Juan, PR 00936-3027

* Corresponding authors.

**Supplementary Information:**

**
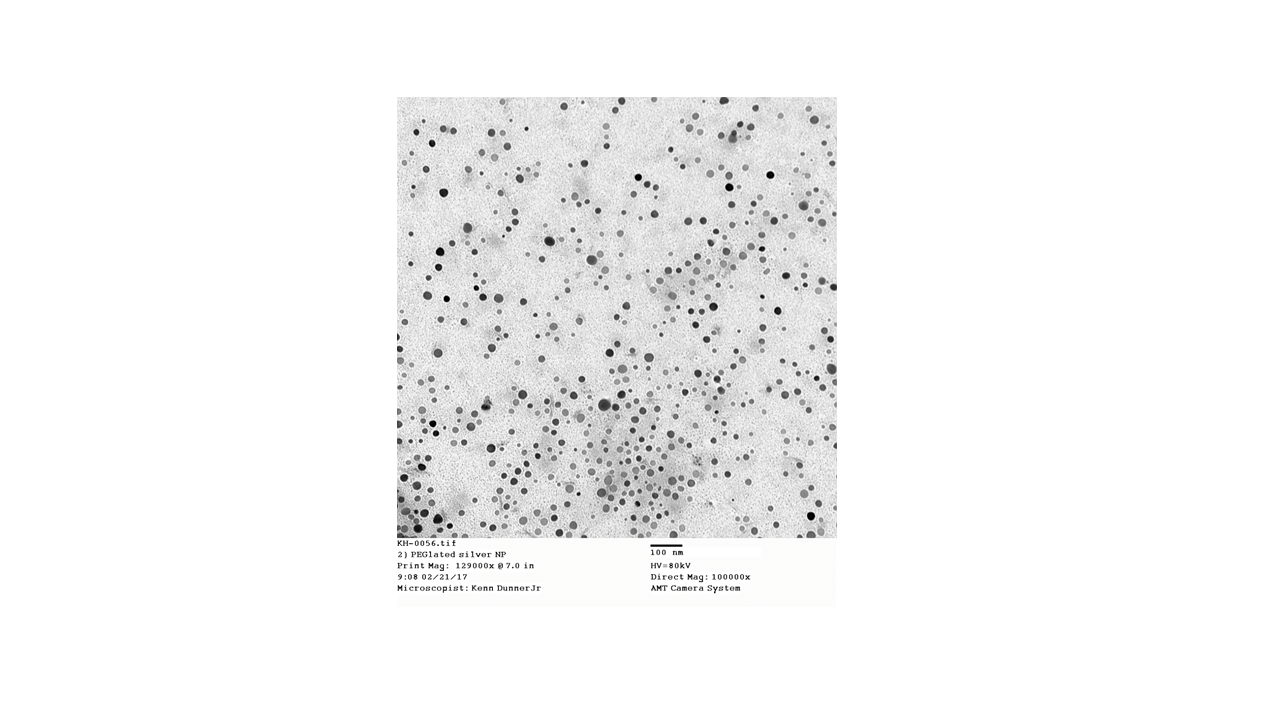
**

**Figure S1**. A) Characterization of pAgNPs using transmission electron microscopy. The image shows that pAgNPs has a quasi-triangular shape after the PEGylation.


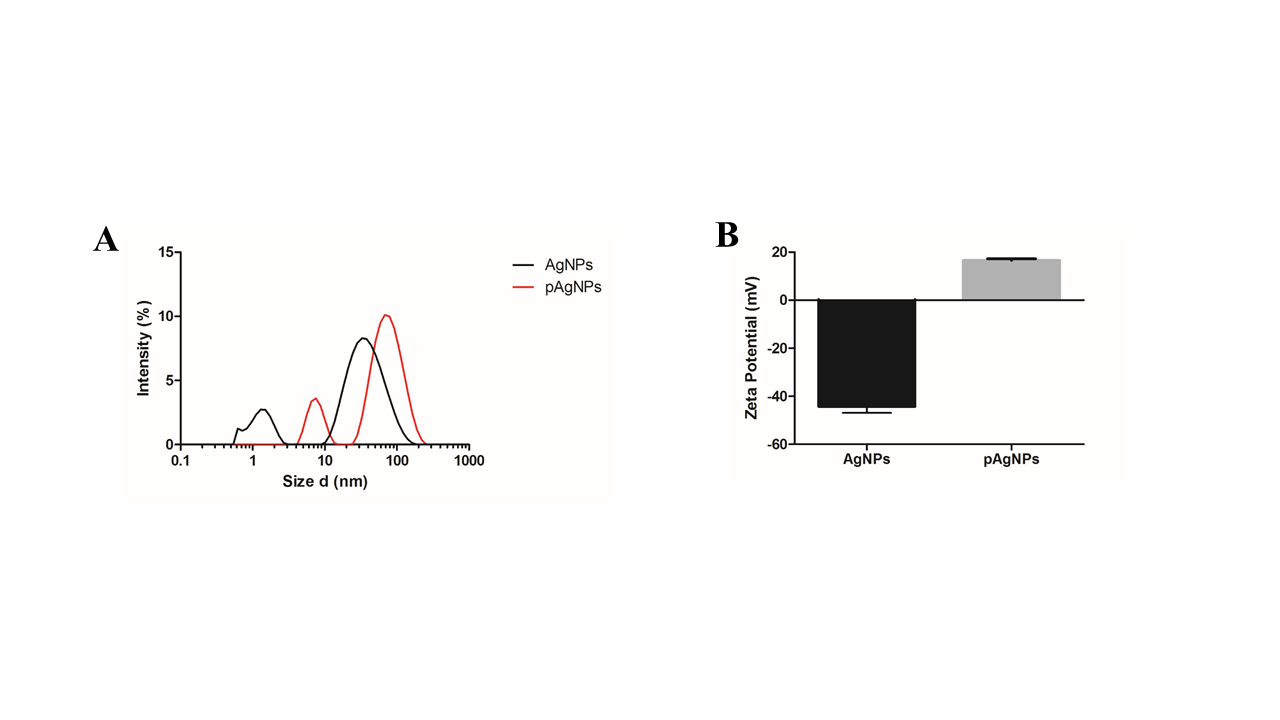


**Figure S2**. A) Characterization of AgNPs and pAgNPs using dynamic light scattering. The spectra show a redshift in the particle size distribution after the PEGylation of AgNPs; D) Zeta potential of AgNPs and pAgNPs, shows an increase in the charge of AgNPs after the PEGylation.


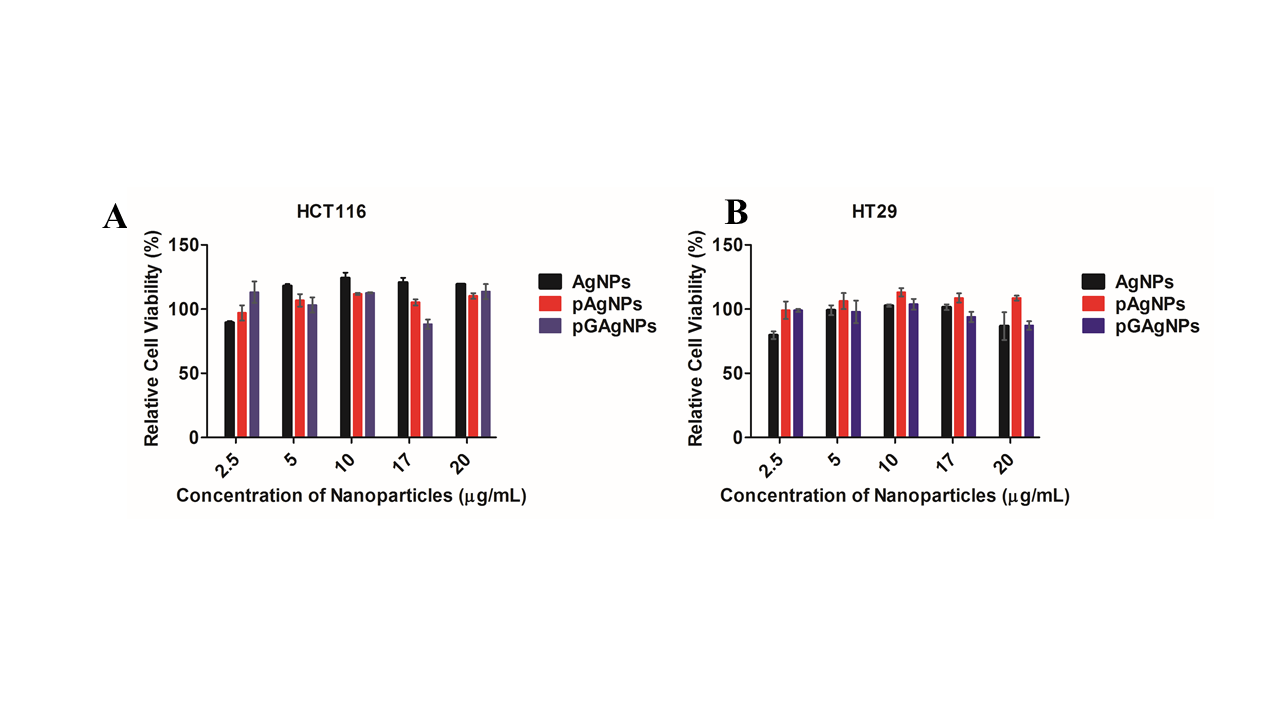


**Figure S3**. *In vitro* Cell Proliferation Assay evaluation of cell viability for HCT116 and HT29 cells treated with AgNPs, pAgNPs, or pGAgNPs. All particles showed high biocompatibility in both cell lines.


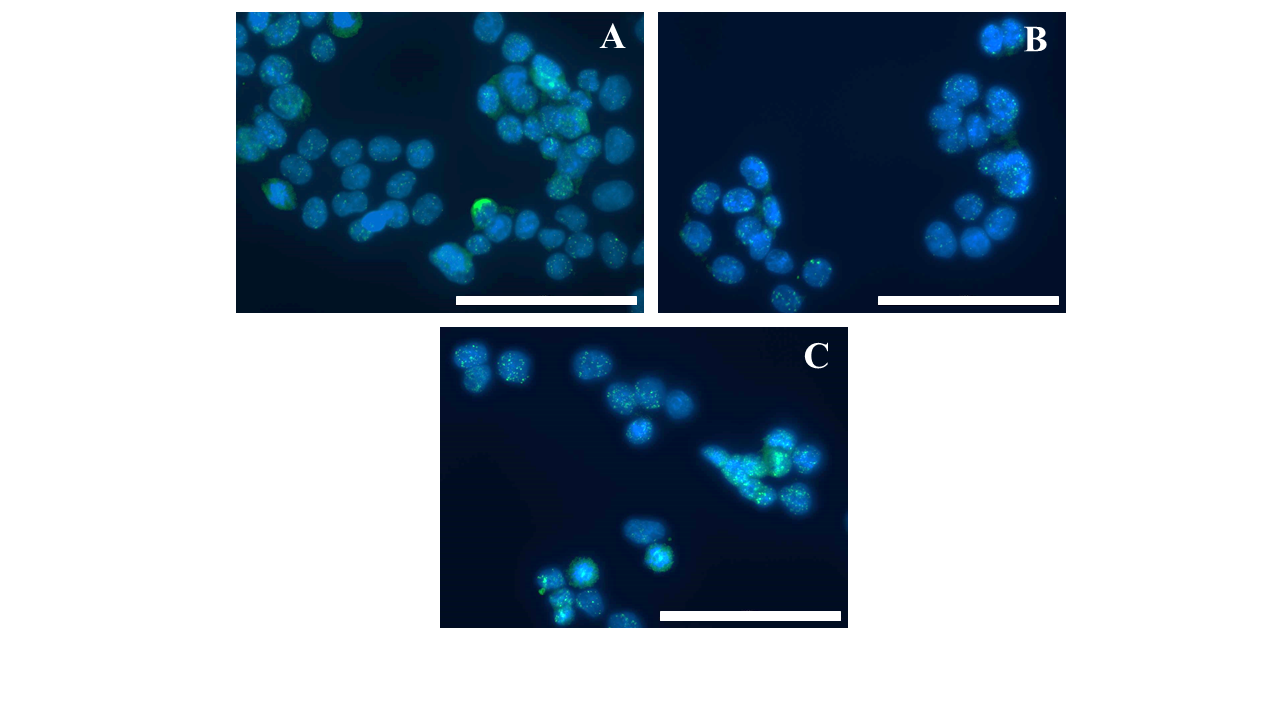


**Figure S4.** 53BP1 immunofluorescence staining of foci of irradiated HCT116 cells treated with A) no nanoparticles, B) pAgNPs, or C) pGAgNPs. At 30 min after irradiation, the pGAgNPs showed more DNA double strand breaks compared to RT alone and pAgNPs. Scale bar is 100 µm.


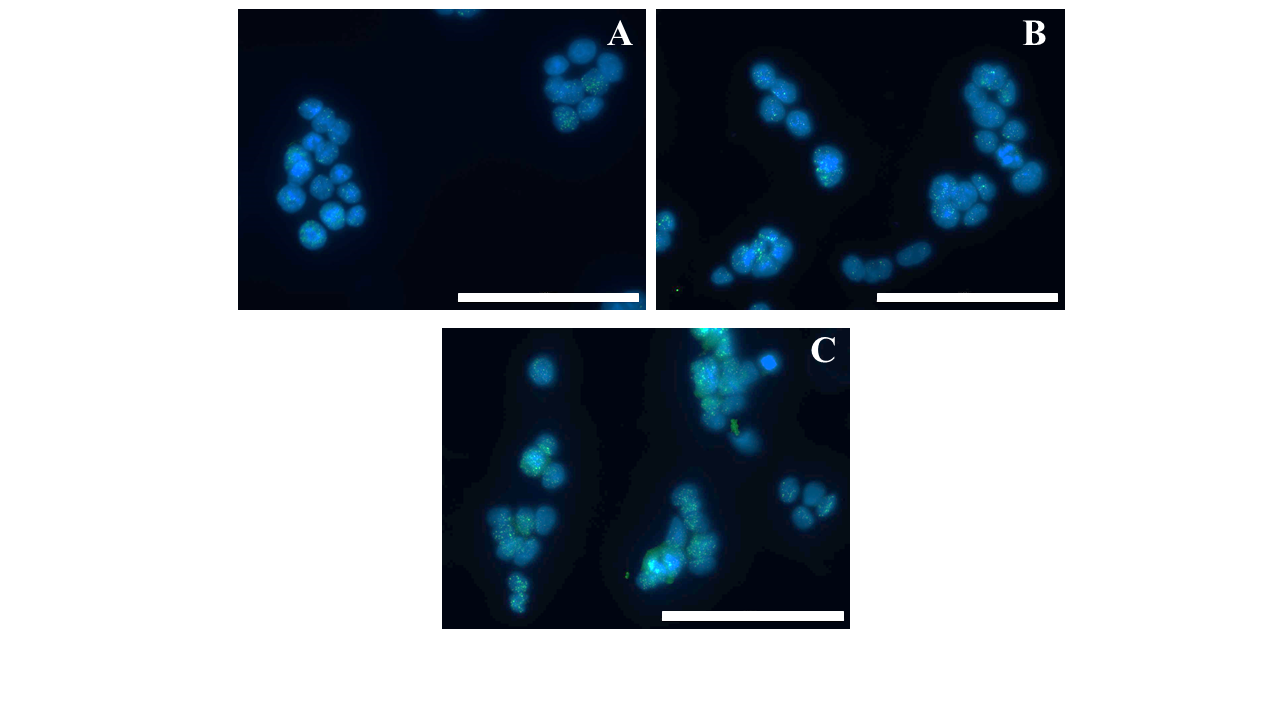


**Figure S5.** 53BP1 immunofluorescence staining of foci of irradiated HT29 treated with A) no nanoparticles, B) pAgNPs, or C) pGAgNPs. At 30 min after irradiation, the pGAgNPs showed more DNA double strand breaks compared to RT alone and pAgNPs. Scale bar is 100 µm.
